# Supplementary figures and images for: Adenovirus Remodeling of the Host Proteome and Host Factors Associated with Viral Genomes
Source: mSystems. 2021 Aug 31;6(4):10.1128/msystems.00468-21. doi: 10.1128/msystems.00468-21 (PMC12338147; doi:10.1128/msystems.00468-21)

Supp Fig 1: WCP quantifies dynamic changes in host and viral proteomes during Ad5 WT infection

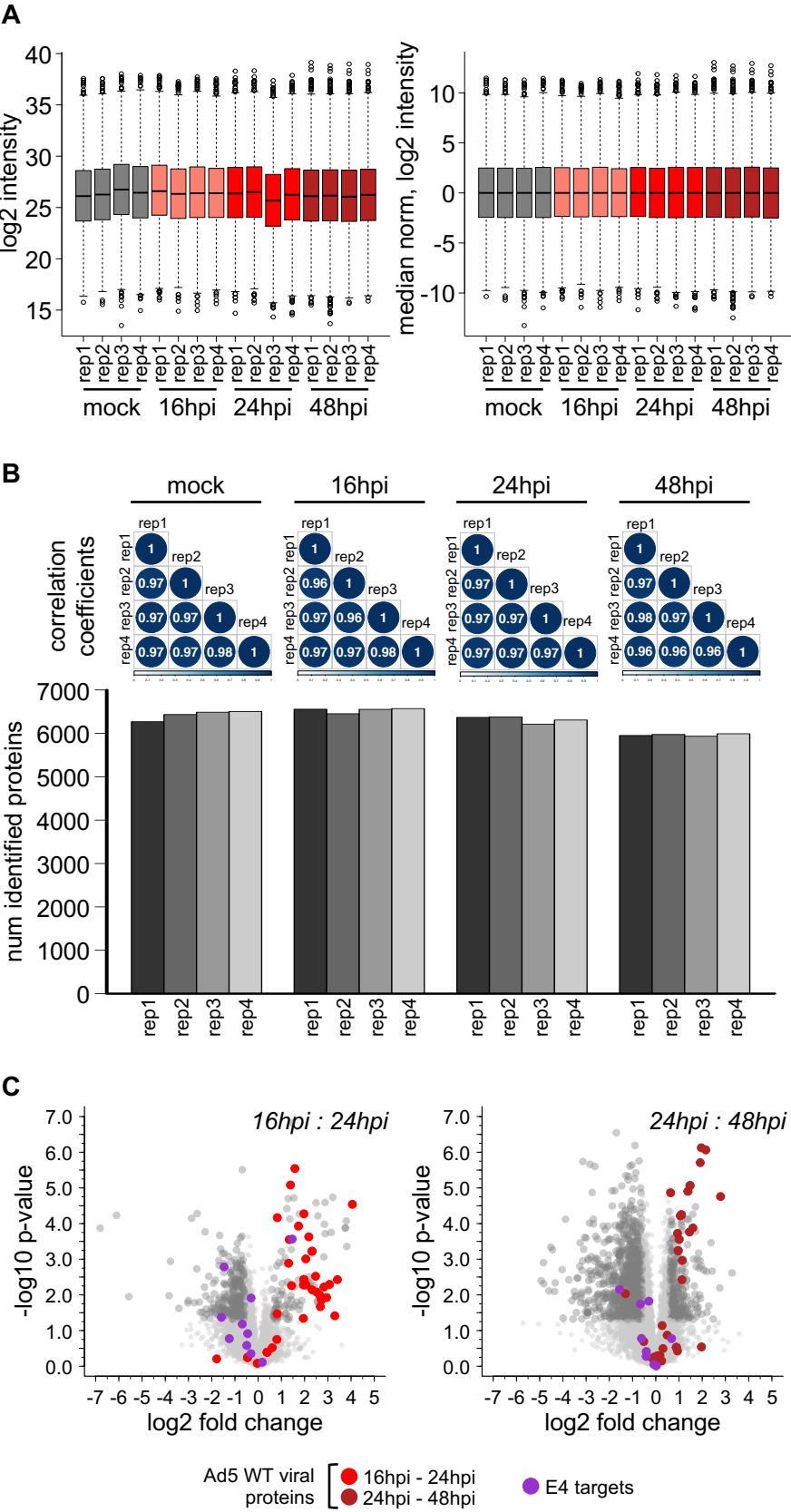

Supplement: FIG S1 [file msystems.00468-21-sf001.pdf]

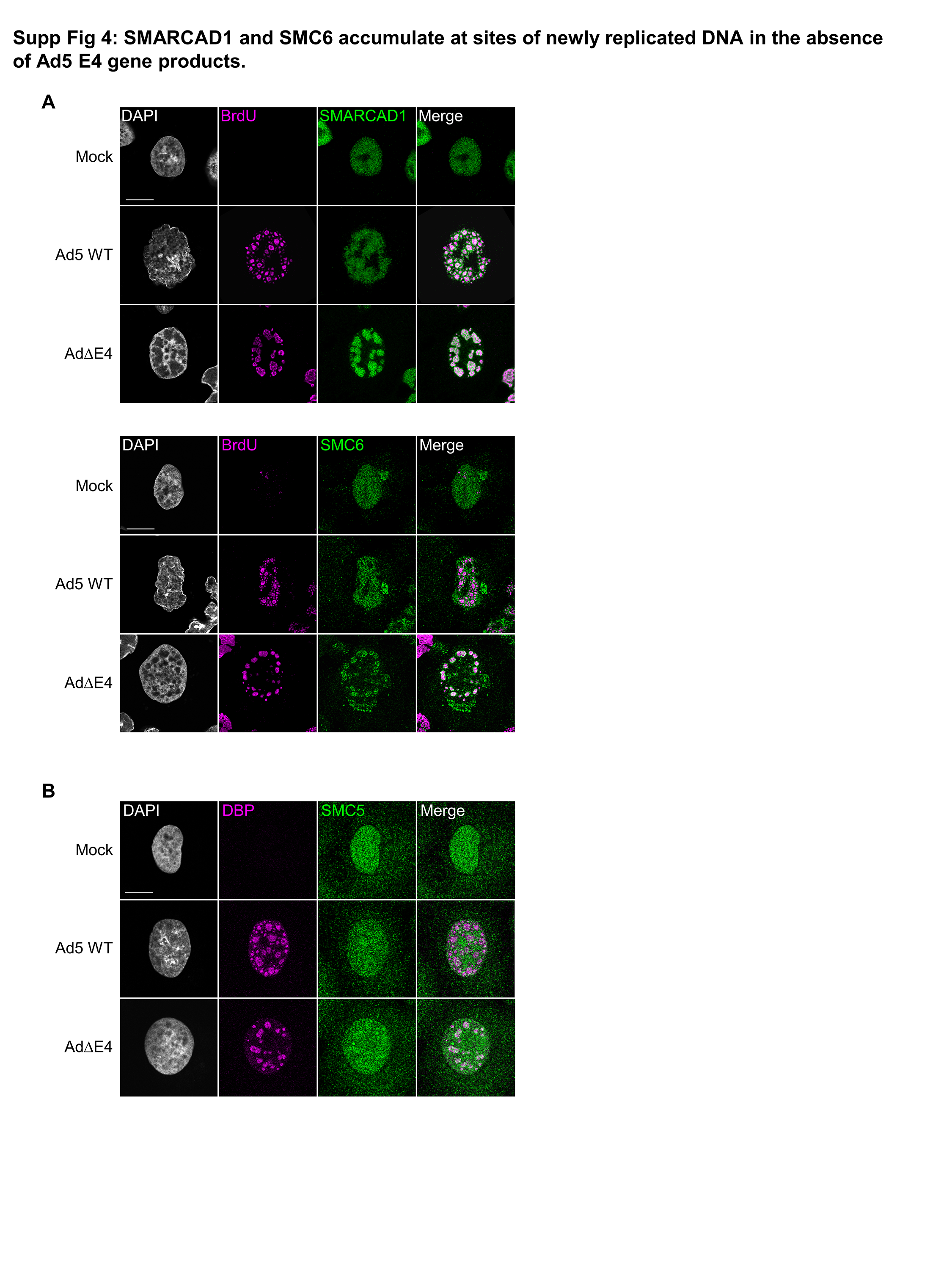

Supplement: FIG S4 [file msystems.00468-21-sf004.tif]

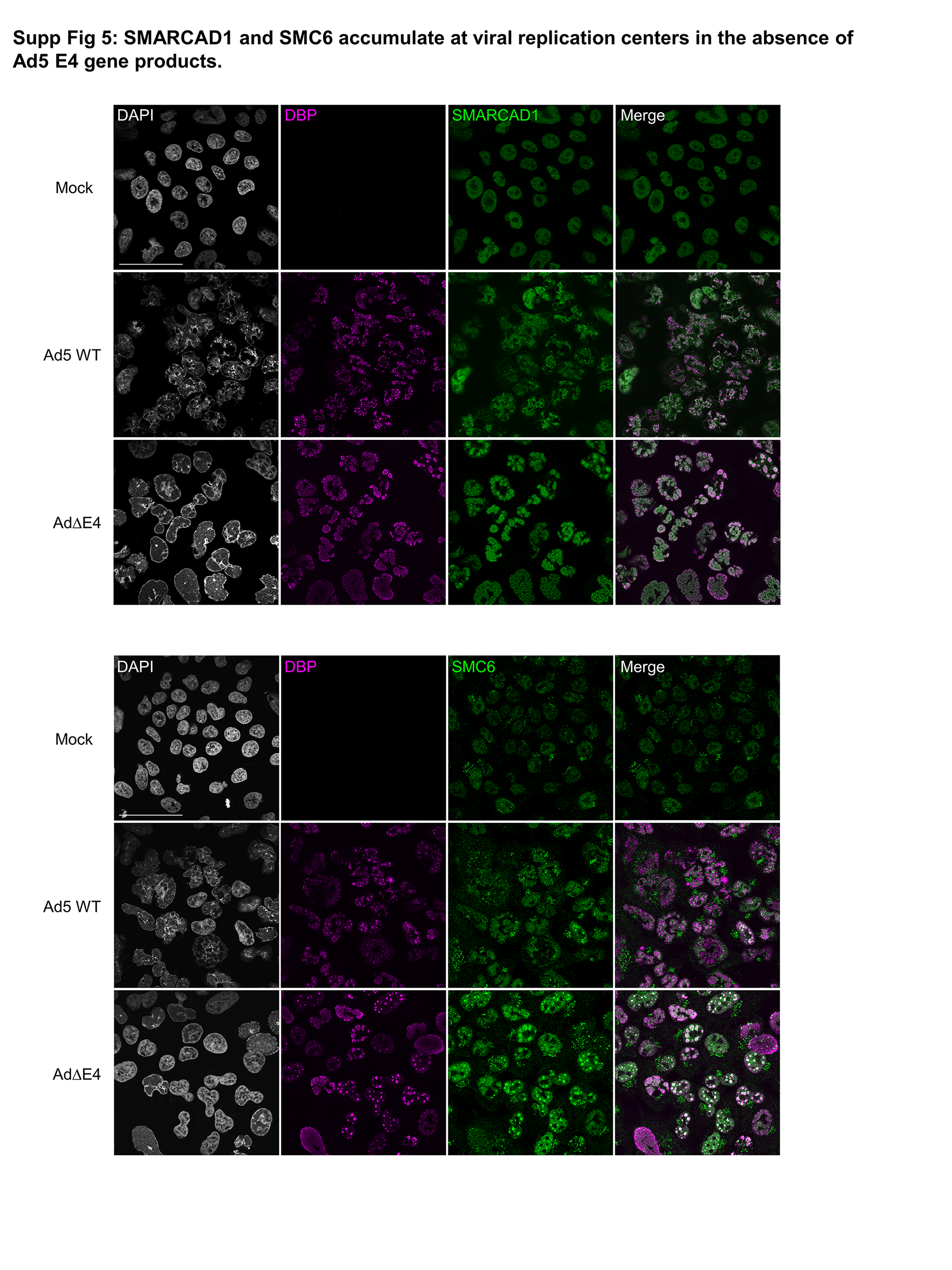

Supplement: FIG S5 [file msystems.00468-21-sf005.tif]

Supp Fig 6: Depletion of SMARCAD1, SMC5, or SMC6 does not impact Ad5 late protein expression.

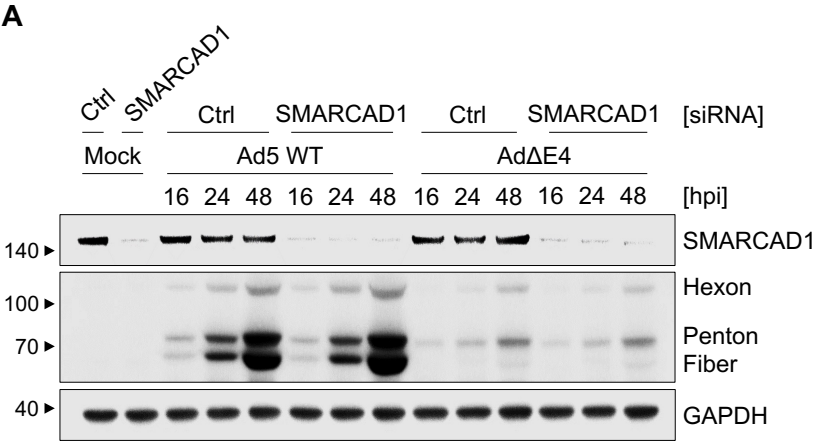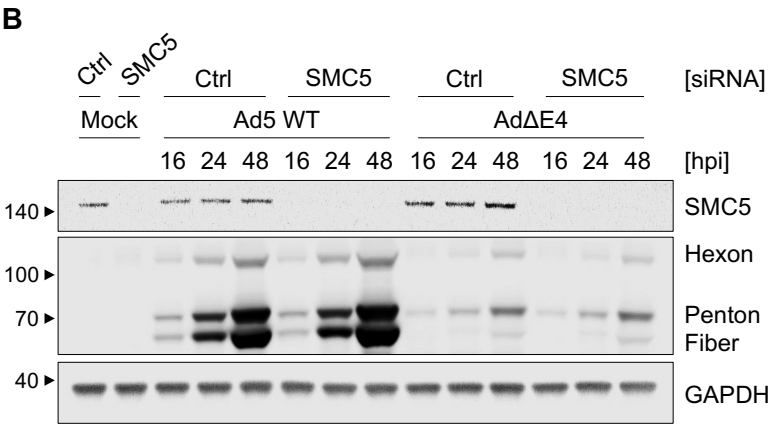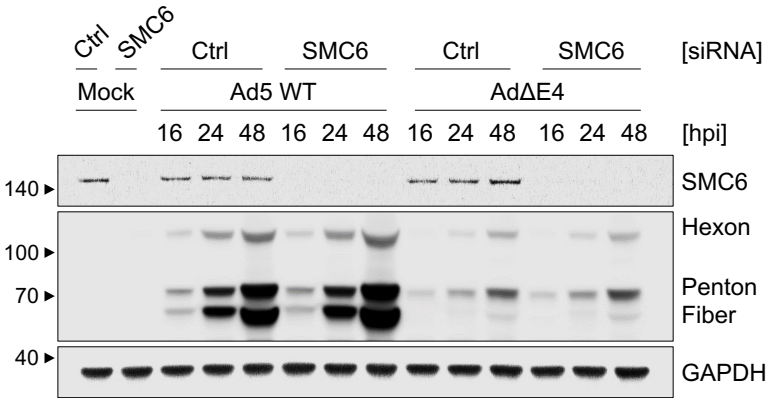

Supplement: FIG S6 [file msystems.00468-21-sf006.pdf]
