# Supplementary material for: Adenovirus Remodeling of the Host Proteome and Host Factors Associated with Viral Genomes
Source: mSystems. 2021 Aug 31;6(4):10.1128/msystems.00468-21. doi: 10.1128/msystems.00468-21 (PMC12338147; doi:10.1128/msystems.00468-21)

Supp Fig 2: Quantification of results shown in Figures 4A, 4C, and 4D.

Quantification of Figure 4A

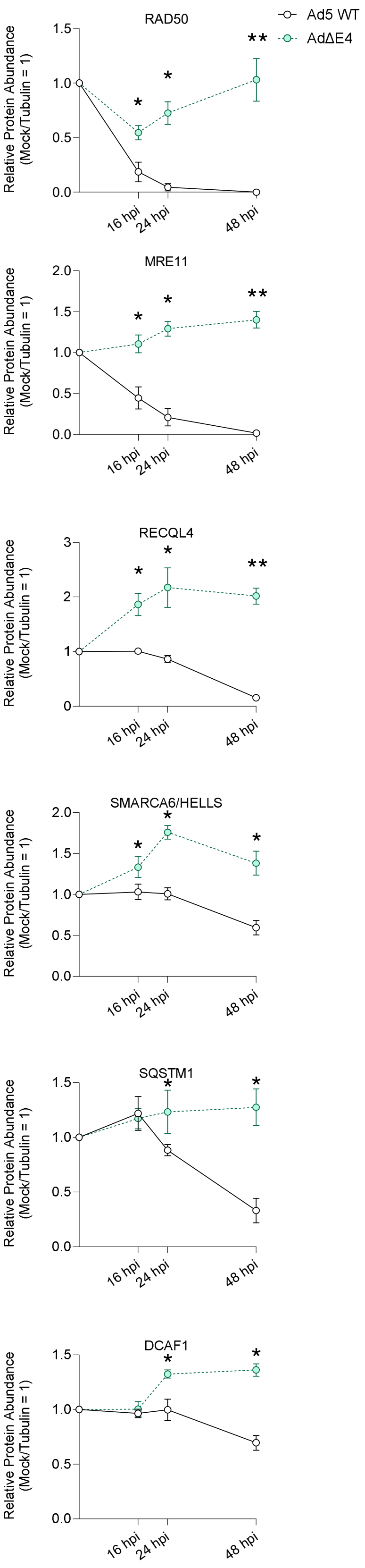

Quantification of Figure 4C

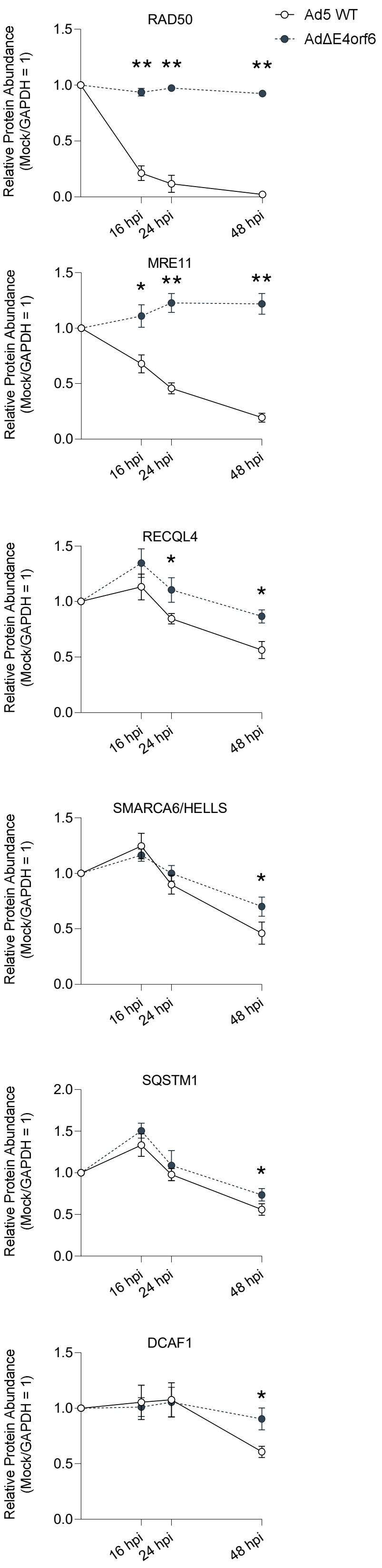

Quantification of Figure 4D

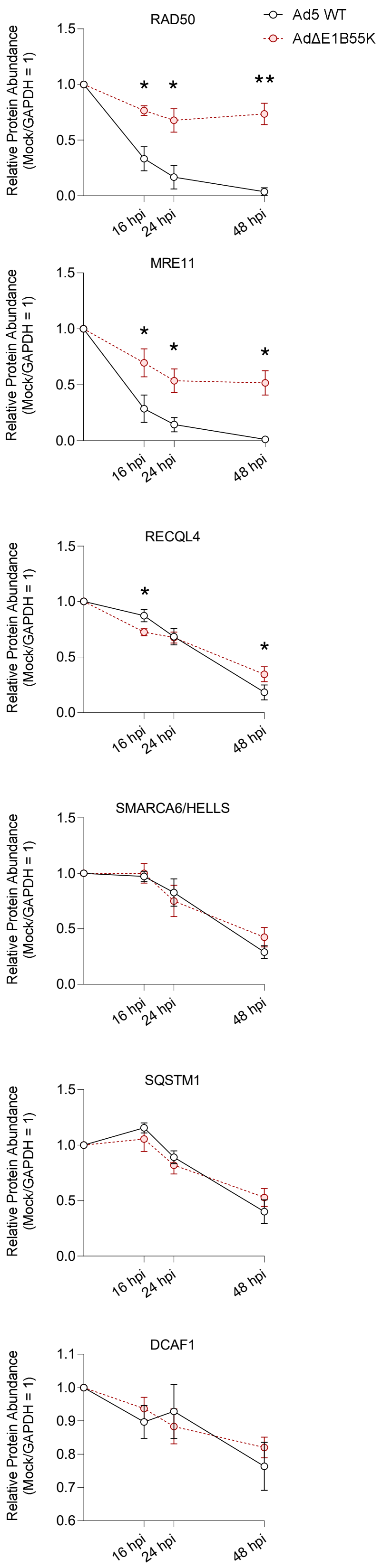

Supplement: FIG S2 [file msystems.00468-21-sf002.pdf]
