# Supplementary material for: Adenovirus Remodeling of the Host Proteome and Host Factors Associated with Viral Genomes
Source: mSystems. 2021 Aug 31;6(4):10.1128/msystems.00468-21. doi: 10.1128/msystems.00468-21 (PMC12338147; doi:10.1128/msystems.00468-21)

Supp Fig 3: iPOND identifies proteins associated with Ad5 genomes in the absence of E4 gene products

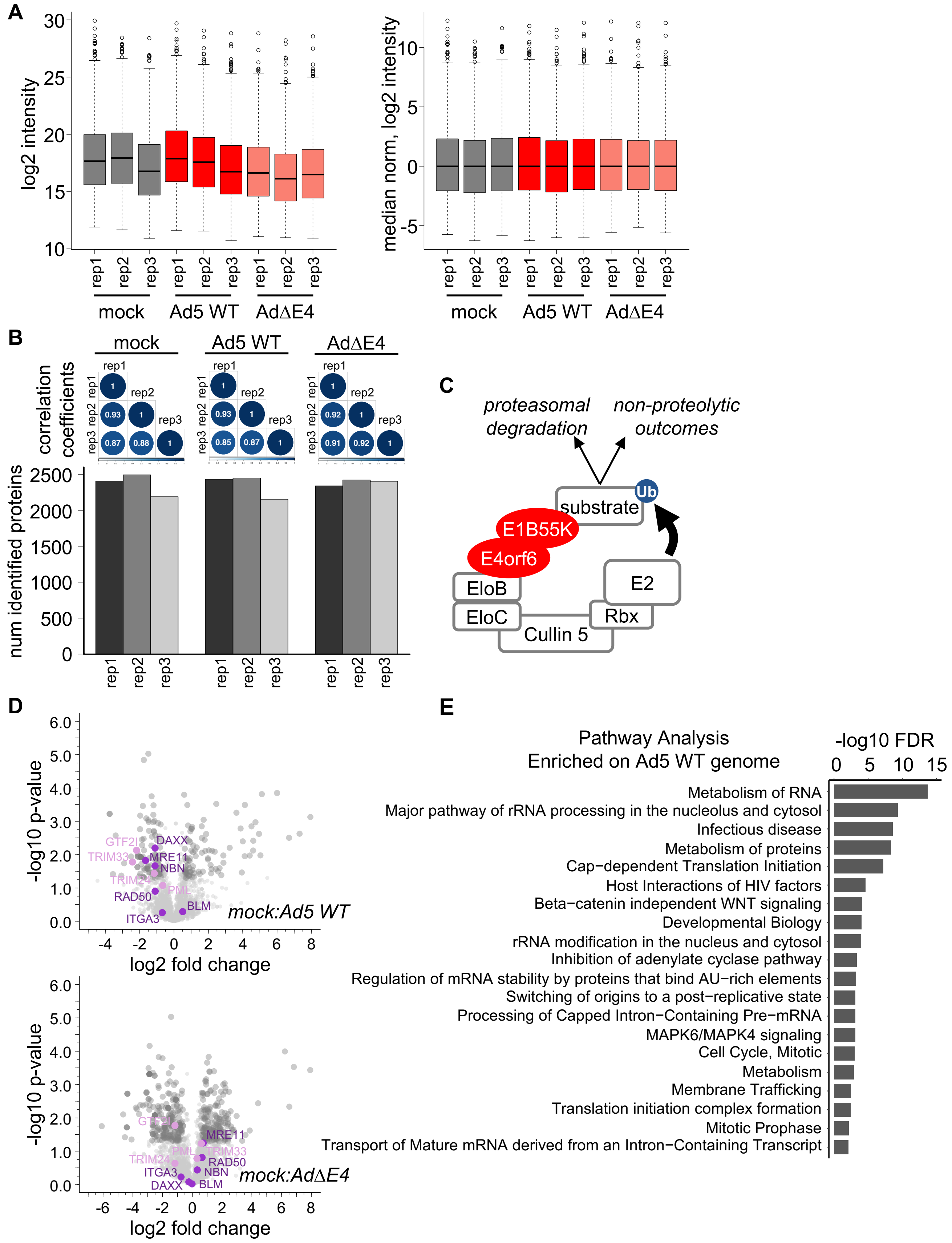

Supplement: FIG S3 [file msystems.00468-21-sf003.pdf]
